# Blood Circulating T Cell Subsets in Relation to Childhood Trauma and Major Depressive Disorder

**DOI:** 10.1192/j.eurpsy.2025.2036

**Published:** 2025-08-26

**Authors:** Z. Susam, A.-L. Boller, J. Freff, T. Kircher, U. Dannlowski, B. T. Baune, J. Alferink

**Affiliations:** 1 Department of Mental Health, University of Münster, Münster; 2 Department of Psychiatry and Psychotherapy, Heinrich-Heine University, LVR Düsseldorf, Düsseldorf; 3 Cells-in-Motion Interfaculty Centre (CiMIC), University of Münster, Münster; 4 Department of Psychiatry and Psychotherapy, University of Marburg, Marburg; 5 Center for Mind, Brain and Behavior (CMBB), University of Marburg and Justus Liebig University Giessen, Giessen; 6 Institute for Translational Psychiatry, University of Münster, Münster, Germany; 7 The Florey Institute of Neuroscience and Mental Health; 8 Department of Psychiatry, The University of Melbourne, Melbourne, Australia

## Abstract

**Introduction:**

Major depressive disorder (MDD) is a prevalent neuropsychiatric condition influenced by genetic, environmental, and inflammatory factors. Alterations in T helper (Th) cell subsets, including Th1, Th2, Th17, and regulatory T cells (Tregs), have been implicated in the immune dysregulation observed in MDD, linking the adaptive immune system to depression. However, the link between these T cell subsets, MDD severity and their connection with childhood trauma (CT), a major risk factor for depression, remain incompletely understood.

**Objectives:**

In this project we characterized peripheral blood T cell subsets and their association with CT and MDD.

**Methods:**

In this study, we performed multiparameter flow cytometry analysis on peripheral blood immune cells from a subgroup of the FOR2107 cohort. T cell differentiation is characterized by their phenotypic markers. Age- and gender- matched groups of 46 individuals with depression and 55 healthy controls (HC) were included, both with and without a history of childhood trauma. Depression severity was assessed using the Hamilton Rating Scale for Depression), (HAM-D21), and CT was evaluated via the Childhood Trauma Questionnaire (CTQ). Correlational analyses examined relationships between T cell subtype frequencies, depression severity, and CT subtypes.

**Results:**

The analysis revealed an increased frequency of circulating Th17 cells in patients with MDD compared to healthy controls. In participants with a history of CT, the overall frequency of CD3^+^ T cells was decreased, while Th2 cells and Treg cell frequencies were reduced when compared to individuals without CT. Frequencies of specific T cell types correlate with CT subtypes, especially in depressive patients. Th1, effector memory T cells (Tem) and central memory T cells (Tcm) showed a positive correlation with physical abuse, while Treg cells correlated with the overall CTQ score and emotional neglect.

**Conclusions:**

Our findings indicate dysregulations of the adaptive immune system in CT and MDD, characterized by alterations in peripheral blood Th17, Th2, and Treg cells. These data highlight the influence of early life adversity on immune function and its potential contribution to the pathophysiology of depression.

**Disclosure of Interest:**

None Declared

